# Exploring the health system response to the COVID-19 pandemic in Cochabamba, Bolivia: a qualitative study with policymakers and managers

**DOI:** 10.1186/s12913-025-13483-1

**Published:** 2025-09-15

**Authors:** Rodrigo Karlop Arce Cardozo, Yercin Mamani Ortiz, Jenny Marcela Luizaga Lopez, Miguel San Sebastián, Frida Jonsson

**Affiliations:** 1 Department of Epidemiology and Global Health, Umea University, Umea, Sweden; 2 Biomedical and Social Research Institute, University of San Simón, Cochabamba, Bolívia

**Keywords:** COVID-19, Policy analysis, Health system response, Emergency, Qualitative research

## Abstract

**Background:**

During the COVID-19 pandemic, health system managers and policymakers were vital in shaping response strategies, allocating resources, and overseeing healthcare delivery. Despite this, limited research has examined their perspectives on the health system response to the crisis, especially in the Latin American context. This study addresses that gap by exploring the health system’s response to this pandemic in Cochabamba, Bolivia, through the lens of these key stakeholders.

**Methods:**

We conducted a qualitative study using semi-structured interviews with 10 health system managers and policymakers responsible for the pandemic response. Reflexive thematic analysis guided the development and interpretation of the themes.

**Results:**

Our findings shed light on how the pandemic revealed and intensified pre-existing vulnerabilities within the health system sectors. Political instability and centralized decision-making delayed the response, increased public unrest, and hindered resource mobilization. Fragmented governance structures and inadequate coordination between the public and social security sectors further weakened the health system’s capacity. Health services, already strained before the pandemic, faced critical shortages in personnel, equipment, and infrastructure. Stay-at-home regulations, short-term staffing policies, and financial barriers exacerbated staff attrition. However, innovations such as telemedicine and non-conventional healthcare strategies helped mitigate gaps in service delivery.

**Conclusions:**

Our findings highlight governance weaknesses, human resource limitations, and structural fragmentation of service delivery, which constrained the Bolivian health system’s ability to respond effectively to the pandemic. Addressing these challenges requires strengthening intersectoral coordination and communication, improving workforce sustainability, and investing in better future public health emergency preparation. Improving governance mechanisms, allocating resources equitably, and integrating service delivery could enhance the health system’s resilience capacity.

**Supplementary Information:**

The online version contains supplementary material available at 10.1186/s12913-025-13483-1.

## Background

The pandemic known as coronavirus disease of 2019 (COVID-19) has profoundly impacted healthcare systems worldwide, exposing vulnerabilities and creating significant challenges for infrastructure, service delivery, and population health [1]. The consequences include increased morbidity and mortality directly due to COVID-19 and indirectly due to disruptions in routine healthcare services. Healthcare systems faced unprecedented strain during the pandemic, which forced rapid adaptations and served as a reminder of the need to build resilient systems capable of addressing current demands and future health crises [2, 3]. Hence, there is a consensus that research is imperative to learn from the COVID-19 crisis in preparing for future emergencies [4].

International studies have explored many aspects of the pandemic, such as health system resilience, governance, and adaptation. Research has shown that policy decisions in response to the COVID-19 pandemic often followed political motives rather than a specific strategy [5]. However, when analyzing government decision dynamics, it is also essential to consider baseline characteristics. These relevant characteristics can include the previously existing centralized or decentralized structures present before the emergency, as described in the findings of positive decentralization processes in three Canadian provinces [6]. Other studies have analyzed the pandemic’s impact on human resources, examining staff burnout, workforce shortages, and strategies such as task shifting, telemedicine, and other digital solutions. In this regard, research has shown that the staffing of health systems is a significant challenge, with a U.S. study suggesting that multiple complex factors contributed to workforce shortages during the pandemic [7]. In many countries, even in developed nations such as Canada, there are different levels of precarity in the pandemic staffing needs of, for example, immigrant staff in nursing homes [8]. Staff attrition resulting from apparently unrelated policies is not only a pandemic outcome; for example, similar effects were also observed in a pre-pandemic US study, where government policies regarding immigrants were identified as one cause of staff reductions [9]. A growing body of research shows disruptions to essential health services and inequalities in access to care during the pandemic. These studies illustrate the importance of sustaining and scaling telemedicine interventions to maintain health programs [10], as well as considering the engagement of the private sector and public‒private partnerships in a comprehensive pandemic response [11, 12].

Regarding the need for research from specific contexts, either by examining countries individually or by region, studies have identified inequities in healthcare outcomes, showing that pandemic interventions, such as mask mandates or gathering restrictions, were associated with reducing cases in the U.S. However, outcomes differ between states [13]. Moreover, even when looking at many countries in a region, such as Africa, differential resilience levels have been identified due to each country’s policies [14]. Unfortunately, there are significantly fewer published studies on the COVID-19 pandemic response in Latin America than in other parts of the world, and many important areas remain understudied [15]. A study of 14 countries in Latin America evaluated each country’s emergency response plans and revealed that the region did not employ a coordinated priority-setting strategy [16]. This highlights the need for thorough examinations of the implementation of COVID-19 pandemic policies in each country, along with their associated health system challenges and outcomes [13]. While this type of research is scarce in the Latin American region, it is almost absent in the Bolivian context, missing a valuable opportunity to evaluate the strengths and weaknesses of specific emergency response policies.

Bolivia is a lower-middle-income country (LMIC), ranking 120 out of 193 countries and territories on the Human Development Index [17], and public health system funding has increased to 4–6% of the national gross domestic product or 10–16% of total public spending once work-based social security is considered [18]. As in many other places in the world, Bolivia’s early response to the COVID-19 pandemic and the first years have been characterized by increased disparities between different healthcare system sectors and their corresponding served populations. By the end of 2021, this country was estimated to have had 419 excess deaths due to the COVID-19 pandemic per 100,000 population, which is superior to most countries in Latin America for the same period [19]. This period was also described as one of increased distrust and despair in the health systems [20]. Nevertheless, while the decision-making processes and resource allocation strategies employed during the pandemic have been examined in other contexts, they remain unexplored in Bolivia [21]. This lack of insight into the barriers and opportunities associated with the emergency response hampers policymakers’ ability to address future challenges effectively. To date, few studies have explored the complex pandemic response in Bolivia. One of them focused on community engagement in a nongovernmental project that was in place during the emergency [22]. In that study, the authors evaluated a health information campaign and the strengthening of the COVID-19 response in a single health center as an intervention project. Their results highlighted the importance of qualitative approaches and the value of cocreation in addressing emerging local challenges. Other qualitative studies have focused on the effects of the forced digital transition of education in this context [23] and the emotional challenges of the general population during this period [24]. However, these studies lack the exploration of health system-wide interventions and focus.

Considering the importance of analyzing strategies implemented during the pandemic from the perspective of those involved in managing the crisis, the current study aims to explore the health system’s response to the COVID-19 pandemic from the perspectives of policymakers and managers in Cochabamba, Bolivia.

## Materials and methods

This is a qualitative study conducted with policymakers and managers of the health system in the Department of Cochabamba who held these roles during the first three years of the COVID-19 pandemic.

### Study setting

Bolivia is a landlocked country in Latin America with a population of 11 million as of 2024 [25]. Geographically and administratively, it is divided into nine departments. Cochabamba is one of these departments; it is located centrally in the country and is home to approximately 2 million people [26]. Administratively, Cochabamba is divided into 16 provinces and 47 municipalities. The province and municipality of Cercado act as the capital for the Cochabamba department, and the peripheral urban metropolitan area surrounding Cercado is formed by four more municipalities: Quillacollo, Sacaba, Vinto, and Colcapirhua [27]. The metropolitan population has 1,239,424 inhabitants, 62% of the department population.

The health system in Cochabamba comprises both public and private sectors, both of which are regulated by the Bolivian Ministry of Health (BMoH**)** [28]. As of February 2019, there was an attempt to establish a universal public health insurance system in Bolivia, serving all otherwise uninsured populations. While promising, the implementation, efficacy, and population reach of the new universal system are still limited [29]. All organizations in both the public and private sectors are responsible for enacting the national policies and strategies established by the BHoM [30]. The BMoH oversees all health services, formulates strategies, and develops plans and programs at the national level, which departments and municipal organizations then implement.

The public sector consists of two subsystems. On the one hand, the public healthcare subsystem includes four third-level hospitals, 18 s-level hospitals, and 470 first-level health establishments [31]. National, regional, and municipal governments fund these services. On the other hand, the Social Security healthcare subsystem is funded through employee contributions and tied to employment. In Cochabamba, there are eight active social security providers, also known as “Cajas” in Spanish, and in 2022, they provided healthcare insurance to 722,812 people or 34.6% of the population in this department [32]. The larger social security providers in this region are the Caja Nacional de Salud (43% of the insured), Caja Petrolera (3%), Caja Cordes (2.6%), and Caja Bancaria (1.2%). Owing to their distinct features, this study treats these two public subsystems as separate sectors.

The private sector includes insurance companies, prepaid medicine companies and services, traditional medicine providers, and non-governmental organizations (NGOs) with diverse staffing and infrastructure management and organization.

### Recruitment and participants

We used purposive sampling to identify and recruit participants, focusing on those who had an active role as health system managers or policymakers during the pandemic emergency period from March 10, 2020, to July 31^,^ 2023, in Cochabamba. We define policymakers as leaders with executive responsibility for the system or sector’s general strategy, and managers with more executive and administrative responsibility to oversee the enactment of these policies at the department, sector, or hospital level, as the lower level of influence. Nevertheless, these levels often overlap, and in quotes, we refer to all of them as managers.

Thirteen participants were identified from different levels of management, representing (1) the national BMoH, Cochabamba health department, or municipal organizations and institutions, and (2) private, public, and social security subsystems. Although the total number of leaders or managers across the various levels and subsystems is not known, the selection process aimed to achieve a balanced distribution across these two dimensions. All the identified health system leaders were invited to participate via an institutional invitation letter. We then reached out over the phone to schedule a brief, initial meeting to present the study and inquire about their interest in sharing their experiences with the pandemic in an interview. The participants who agreed to participate in the study were scheduled for a physical meeting to provide informed consent and conduct a semi-structured interview. In total, 10 managers and policymakers participated, with 2 being women and 8 being men. Their ages ranged from 44 to 73 years. All were medical professionals with a level of education equivalent to a master’s degree or a doctorate in health-related fields (e.g., public health, health system management) and with experience in relevant leadership roles starting at 6 months and up to 30 years in the Bolivian health system, which is in addition to the minimum entry requirement for leadership roles in the health system being 5 years of professional experience. However, during their interviews, almost all of them were no longer in the specific roles they had taken during the health system emergency due to the constant rotation of health managers in all subsystems in Cochabamba and Bolivia.

### Data collection

Our data were collected through semi-structured interviews conducted between May 15, 2023, and March 28, 2024, with seven interviews taking place in 2023 and three in 2024. The last three interviews aimed to increase the representation of subsystem voices and women. The interview guide was developed based on inspiration from a similar study conducted with policymakers in Nepal [33]. However, to adapt the guide and test it in the Bolivian context, we first conducted a pilot interview in Spanish with a previous health system manager of Cochabamba. After this pilot, we made minor adjustments to the guide, adding an exploratory section to capture areas or aspects of the response that had not yet been identified. The resulting interview guide was approved by our ethics committee and used in the data collection.

The semi-structured interview guide developed for this study (Annex 1) consisted of open-ended questions organized into six thematic areas: (1) overview of COVID-19 public health management; (2) characteristics of the Bolivian health system before, during, and after the pandemic; (3) allocation of human resources during the pandemic; (4) allocation of equipment/resource management (space, equipment, and facilities) during the pandemic; (5) recommendations and ways forward for the health system; and (6) other areas or aspects of the response.

The first author conducted the interviews in Spanish, the participants’ native language, either in a private room at the Biomedical and Social Research Institute (IIBISMED, after the institute name in Spanish) of San Simon University or at the participants’ offices. The interviews ranged from 47 to 90 min, were audio recorded with participant consent (Annex 2), and were transcribed verbatim in Spanish, with all personal identifiers removed.

### Data analysis

Data was analyzed using reflexive thematic analysis [34]. The data collection was conducted in parallel with the initial analysis and continued until enough data had been collected to address the study aim. We reached this conclusion of analytical sufficiency, in line with the constructivist and interpretive, reflexive thematic analysis [35]. The analysis was conducted in two stages, where the first was inductive and data-driven. We read the emerging transcripts and compared them against the voice recordings. Three transcripts were translated into English for discussion within the research team. Initial thoughts and ideas were written down as analytical memos, generating a list of focus areas to explore. This first stage also served to identify the health system dynamics framework as a relevant analytical lens [36].

In the second stage, following the steps of Braun and Clarke [34], the data analysis was deductive, with the transcripts being analyzed through a lens of the health systems dynamic framework. The data were divided into meaning units with a coherent message as we analyzed the texts, assigning the extracts with interpretive codes. This initial coding was performed by creating single or multiple codes for each meaning unit of transcripts. After this, the codes were grouped with others of similar meaning and sorted into potential themes and subthemes in an iterative process of thematization focused on the research question and the health systems’ dynamic framework. This process was then reviewed and discussed, with the thematization being revised and continued by considering similarities and differences across codes, subthemes, and themes until they formed a coherent pattern. The final step consisted of defining and naming the themes and subthemes while writing the report to tell the story of the data in depth [37].

## Results

We developed three themes based on the analysis: (1) top-down centralization, evolving toward decentralization and coordination; (2) experienced human resources were missing when most needed; and (3) service delivery challenges spurred innovation and the use of nonconventional treatments. These themes are described below, along with quotes from participants.

### Top-down centralization, evolving towards decentralization and coordination

The first theme captures governance responses to the pandemic. These responses focused on containing disease transmission, using rigid community quarantines and a centralized approach to COVID-19 services. While initially successful, these strategies eventually failed to prevent community-wide disease transmission. They also had unintended consequences and were progressively replaced by a more decentralized approach.

As described by our participants, the initial health system response to the pandemic in Bolivia focused on case containment. To achieve this, the strategy included strict community quarantine nationally, within departments, and in cities. These quarantine measures were combined with border closings to other countries. Bolivia was one of the first countries in Latin America to enact and maintain close border policies and strict quarantines, which lasted from 1 to 3 months. One participant explained:

*It was the first country [Bolivia] to close its airports in all of South America. (…). It was the first country that confined [the population] and said*,* ‘We are going to stay at home’ (…). I think it is one of the more successful measures that existed.* Manager 3.

The participants stated that when the first cases of COVID-19 were confirmed in March 2020, the health system’s response, following the community quarantine and border closures, was to centralize care for suspected COVID-19 patients and isolate those with confirmed positive PCR test results. However, according to the participants, this was difficult to implement fully due to a lack of compliance within the population. In response, the quarantine was enforced by police and military personnel, who conducted patrols on the streets and coordinated efforts to prevent and punish noncompliance, with fines or jail time as penalties. As narrated by one of our participants:

*There are towns [referring to peri-urban and rural towns] that refuse to have biosecurity and isolation. We had to take the Army*,* as in dictatorships*,* because people did not understand.* Manager 3.

In the context of post-election changes in leadership due to political disputes at various government levels, the centralized response to COVID-19 services involved several stages. It initially focused on testing and caring for suspected and confirmed COVID-19 patients, as well as conducting contact tracing and implementing isolation measures. The participants described how first, only one reference laboratory was available for PCR testing in the country, which meant that *“[when] the sample was taken*,* it was first sent abroad*,* outside of Bolivia*,* and later it was sent to INLASA (national reference lab) La Paz and Santa Cruz*” (Manager 5). Testing was later expanded to one PCR-enabled laboratory per department. In this stage of regional centralization, each department also designated one hospital for COVID-19 treatment and one quarantine center to isolate recovering patients and asymptomatic suspected cases. However, most participants described these approaches as limited or insufficient once community transmission was established.

*Because*,* at the beginning*,* everything was centralized in the SEDES laboratory*,* the CENETROP laboratory*,* and the INLASA*,* and everything had to be sent there. And obviously*,* that is a very heavy burden in a critical situation. We experienced that. The moment they [the BMoH] authorized us*,* we started to see that the laboratory X*,* Z*,* were enabled to do PCR*,* we immediately made agreements and already had access to the diagnosis.* Manager 1.

It is essential to note that our participants described the Bolivian health system as being saturated and fragmented before the pandemic, with multiple public sectors facing substantial strain. Given this low pre-pandemic baseline capacity, the different stages of centralizing services, both national and regional, were utilized to better deliver essential care to individuals diagnosed with COVID-19. However, when the number of cases continued to rise, the centralized COVID-19 services became scarce, resulting in insufficient capacity and delays. As described in the case of COVID-19 test results, one participant explained:

*In the larger cities of Bolivia*,* Cochabamba*,* La Paz*,* Santa Cruz. (…) Your diagnosis took at least 4 days because we all sent it to INLASA [the first reference lab] in Santa Cruz. Now*,* you talk about Potosí*,* Oruro*,* and Tarija [examples of smaller cities]*,* where do they send it*,* [the samples to be tested] that is*,* and it was tremendous.* Manager 7.

Posterior to these issues, participants described how departmental and municipal governments were given greater autonomy in addressing their specific needs. This process requires that all healthcare services care for both COVID-19 patients and non-COVID-19 patients, resulting in a forced expansion of service delivery capacity, testing, and isolation for all sectors. During this decentralization process, our participants described how a collaboration strategy emerged in Cochabamba, known as the “situational room,” which became essential in managing the pandemic. This space was developed to provide a platform for all levels of government, health system subsectors, community representatives, and media to participate and collectively determine the best responses for all sectors.

*It was a situation room that also allowed us to unite not only the three health systems that we had*,* but also the population (…). The situational room was also essential for dealing with it here*,* in Cochabamba*,* especially on how to unite everyone*,* so that everyone has a single idea of what was happening.* Manager 5.

Prior to this strategy, political disputes among different levels of government and frequent leadership changes hindered coordination between health system sectors. The situational room, therefore, served as a strategy to support departmental decision-makers by providing technical and managerial guidance and creating a space for collective organization and coordination. The participants also described it as a space free of political discussions:

*We did not talk about what we should have talked about*,* which was health. So*,* in the situational room we did*,* and it stuck. I remember*,* there at the door*,* and it said*,* “Here we only talk about health”*,* we do not care. Reds*,* greens*,* yellows*,* grays. We do not care who. We only come here [the situational room] to talk about health.* Manager 5.

However, the situational room was temporary. One participant explained that it was eventually discontinued approximately six months after its establishment, as the pandemic was controlled, and that there was no legal framework for its continued existence. The participants also described how it resurfaced when needed, as in the 2023- 24 dengue outbreak case.

### Experienced human resources were missing when most needed

This second theme describes responses to the pandemic, which focused on consequences for human resources. It includes policies that allowed healthcare workers in high-risk groups to stay home, which contributed to staff shortages that exacerbated preexisting resource gaps. It also contains descriptions of a short-term hire program that temporarily filled the void created but proved unsustainable in the long term.

As some participants mentioned, the inadequate quantity of healthcare workers was a critical problem in Cochabamba before the pandemic. This deficiency was primarily driven by a lack of financial support to fund hiring these workers in the public sector, rather than a lack of availability. With this background, regulations from the national government, described by our participants as a reaction intended to protect “high-risk” individuals, were rapidly introduced at the beginning of the pandemic. These regulations allowed healthcare workers, many of whom had extensive experience and specialized training, to stay at home while continuing to receive their regular salaries. As a result, many public and social security staff members withdrew from frontline service delivery. While one participant narrated how “*the generals*,* who should be commanding the battle*,* were suddenly taken away from us (…)”* another one explained that:

*The older human resources. We are talking about 50 [years old] and up. Did not want to answer*,* many took refuge in a Bolivian law that come out*,* I do not remember*,* right now I do not remember the number of the law*,* but where it says: if you are over 60 years old as a man and over 58 as women*,* you can take refuge and leave to your home and do remote work. If you have children under 5 years old*,* you can go home. If you are a pregnant woman*,* you can go home. If you are the husband of a pregnant woman*,* you can go home. So*,* the hospital was left with 50% of its employees.* Manager 2.

Several participants also mentioned that many health professionals who subscribed to these ‘workers under high-risk’ policies in the public sector continued providing services in the private sector, where working conditions or remuneration were better. They further explained that the funding and labor law restrictions in place before the pandemic created a regulatory framework that reduced managers’ capacity to hire staff in emergencies, such as the pandemic. The reaction of the government to this roadblock was to enact regulations allowing for temporary, short-term hiring of the much-needed health staff:

*In the context of the pandemic*,* a new budget was drawn up to hire doctors. So*,* that was a very important measure. Having already decreed an emergency law that allows the temporary hiring of health personnel. Because otherwise*,* we would not have been able to contain it.* Manager 3.

While these new regulations allowed three-month short-term contracts, this hiring approach was only partially effective. As described by our participants, staff sometimes arrived at the end of a surge in cases, or their short contracts ended just before a surge occurred, necessitating multiple cohorts of short-term hires. Ultimately, the brief nature of the programs did not create a sustainable approach to hiring staff during the pandemic emergency, as explained by one participant:

*The first people with these contracts*,* contracts that they called ‘temporary’ arrived (…) when the first wave was already going down (…). Then everything went down; let us say everything was contained because it lasted almost four or five months*,* the first [wave of cases]. We had already contained everything*,* and the hospital was full of people just walking around. And they finish their three months and leave. And the second wave began*,* which was supposed to start strong*,* but it did not start. So*,* for the third wave*,* they say since there was no second wave*,* there was not much effect*,* we are not going to hire them anymore*,* and the third wave came with force.* Manager 7.

### Service delivery challenges spurred innovation, and the use of nonconventional treatments

This third theme focuses on responses to the pandemic related to service delivery and the many health service limitations during the COVID-19 surges. It includes descriptions of deficiencies in supplies for treatment and protective equipment created by the pandemic, which were partly overcome or addressed through innovation or the expansion of nonconventional service delivery.

Following the decentralization stage, the increased demand for services compelled health sector managers to allocate more space for outpatient and inpatient services, as well as intensive care units (ICUs). Private spaces, such as hotels, pre-pandemic outpatient services, and administrative offices, or even building temporary structures for quarantine, inpatient care, or intensive care units (ICUs), were used to overcome this situation. As explained by one participant:

*So*,* what has been done in emerging countries like Bolivia? They hired a hotel to admit patients of medium complexity*,* and even the serious ones. They were supposed to be taken to the hospital*,* but the hospital did not have space*,* so that is what we did.* Manager 2.

Even if the participants described how the saturation of services existed long before the pandemic, vast differences in resources and response capacity among the sectors surfaced. This was evident when the participants discussed how smaller but richer subsectors rented or purchased space from the private sector when needed, compared to larger but less affluent subsectors that could not and were overwhelmed.

*We all thought that we were going to have difficulties. But I think that*,* rather than that*,* our work model*,* the management model that we have*,* has allowed us to go to the different private clinics that offer hospital care and intensive care. And something that we are proud of in the regional offices is that not a single patient of ours has died or had a bad outcome because we did not have a hospital bed or an ICU bed.* Manager 1 (in a small but well-funded “caja”).

*The hospital was overwhelmed; there was nowhere to isolate them [COVID-19 positive patients] because the unions*,* the trade associations*,* and everything else [other organizations] were putting so much pressure on them.* Manager 10 (in a large but under-funded “caja”).

The government introduced regulations allowing pandemic-related acquisitions that provided some flexibility in purchasing resources such as equipment and medication, but not in building new infrastructure. These regulations became barriers that the public and social security sectors overcame through innovation, creating so-called “domes” or other non-brick-and-mortar temporary space capacity. The participants referred to how some subsectors built these spaces in collaboration with private companies as an example of public‒private partnerships.

*The characteristic that this dome has is that it is completely removable. No*,* it is not a building*,* it is an object that the institution can dismantle at any time if it wants and move it*,* but included everything: central oxygen*,* aspiration*,* vacuum*,* and a special background ventilation system to facilitate given the magnitude and size of the virus to facilitate aspiration.* Manager 2.

The participants also consistently mentioned how the lack of personal protective equipment (PPE) and other crucial materials fostered local innovation. This created space for local public and private partnerships to produce equipment and supplies, rapidly accelerating local production. Personal protective gear, such as masks, hazmat suits, as well as critical medical devices like continuous positive airway pressure (CPAP) parts and ventilators, have been developed locally. Under normal circumstances, these advancements might have taken much longer to achieve, if at all, but the urgency of the situation forced this rapid adaptation response.

*I mean*,* it was not even enough for one hour [talking about PPE]. So*,* we did not have supplies*,* of course*,* we did not have boots. Do you remember the photos of Bolivia that circulated in the world? We would put these black plastic garbage bags on. A major problem is that the situation that occurred also led to the distortion of the biosafety teams. You may have seen ‘Aguayo’ [a traditional fabric used in industry and clothing] for a mask…. The biosecurity issue was not well managed.* Manager 3.

In addition, healthcare workers were often forced to improvise, using makeshift alternatives such as plastic bags for boots and extending the use of PPE far beyond recommended guidelines. The roots of these limitations were based on a reliance on imported goods and the resulting limited domestic industrial production:

*We do not produce medical equipment; we import it all*,* and the little that could arrive came from Brazil or Argentina*,* because to think of Europe. No*,* there was no possibility.* Manager 2.

Ultimately, the innovative efforts helped alleviate immediate shortages and highlighted Bolivia’s potential to overcome its dependence on external supplies despite geographic and industrial limitations, with innovation and partnerships with local businesses.

*We began to adapt all of this [referring to items used for ventilator support] here in Cochabamba*,* the biomedical doctors (…) they began to make equipment “Made in Here” that’s what they are called*,* which began to function.* Manager 5.

As a contextual element in this setting, Bolivia has a long tradition of using telemedicine, with several programs introduced in the public sector in the last 15 years. Despite this, the general population’s adoption of telemedicine was relatively low before the COVID-19 pandemic. Additionally, Bolivia’s healthcare landscape includes a significant traditional medicine sector. During the pandemic, the demand for telemedicine and traditional medicine increased as healthcare services were overwhelmed. One strategy highlighted by the participants was the use of mobile applications, such as the one called “Health Cochabamba.” These applications facilitated telemedicine consultations, helped triage patients, and guided people on whether they needed further medical care. Many of our participants mentioned these innovative experiences, which were led primarily by the public sector with support from private partners.

*Well*,* there has always been a lack of human resources; that is*,* there have never been enough. But this strategy. To use technology*,* this application*,* this so-called “system”. It has worked. A huge number of people have attended to us*,* and many of them have also called us. They thanked us because it was not just a one-time call*,* but (…) we have a protocol to call back every 3 days*,* especially those who were positive.* Manager 4.

In the case of traditional medicine, health system leadership officially encouraged the population to use it. However, our participants explained that this period also saw a rise in questionable nonconventional treatments driven by misinformation, where remedies such as ivermectin, a veterinary antiparasitic agent, and chlorine dioxide, an industrial chemical, gained popularity despite a lack of scientific support or traditional use. In some cases, even healthcare professionals turned to these treatments, as described by one of the participants:

*This is the solution to the whole world. The medication*,* the preventative*,* is the horse dewormer. The Ivermectin. I also gave the staff Ivermectin*,* I did give it to them [hospital staff]*,* ‘here it is’ if you like you can take*,* if not [implying they had an option not to take it]*,* but as an authority I suggest you. I think it worked; we did a kind of monthly deworming with Ivermectin.* Manager 2.

## Discussion

In this study, we explored the health system’s responses to the COVID-19 pandemic in Cochabamba, Bolivia, from the perspective of its health system leaders. The results illustrate the governance of the crisis response, highlighting rigid strategies such as community quarantine and service centralization as the basis for initial disease containment. We also identified specific human resource policies implemented by the Bolivian government during the pandemic, which seem to have increased staffing attrition, contributing to workforce shortages. Innovation in service delivery strategies has focused on the development of new public‒private partnerships, as well as the expansion of existing telemedicine and traditional medicine sectors. Overall, the findings offer valuable insights into the health system strategies employed in response to the COVID-19 pandemic in Bolivia, which are crucial for informing future emergency responses in the country.

Like other parts of the world, Bolivia implemented strict community quarantine measures to manage the spread of COVID-19. These measures had significant economic and social consequences. They were difficult to enforce, prompting the military and police to execute and uphold them; the resulting increasing social unrest is consistent with reports from China [38, 39]. In Bolivia, the initial centralization and rigid pandemic response eventually shifted to decentralization and increased coordination between health system sectors and society. In other contexts, the health system’s political and structural factors influence whether centralization or decentralization is used as a response during a crisis. This was described, for example, by Greer et al. [5], who report that credit or blame for centralization or decentralization in Austria, Czechia, and France followed political motives. In addition, Smith et al. [6] has described how the previous state of decentralization or centralization of regional health systems in Canada was associated with the decision of the direction of a response strategy toward one or the other. Notably, during the pandemic, Bolivia experienced a significant political crisis, coupled with an economic crisis and persistent social unrest [40]. This influenced the decision-making process regarding measures to respond to the pandemic and created differential outcomes at the subnational level. However, actual political motives were more complex to elucidate through our interviews alone, and we believe that they should be explored in more depth in future work. Furthermore, the rigid strategies employed during the initial COVID-19 response do not appear to have been accompanied by efforts to strengthen the private, public, and social security health sectors in preparation for the future decentralization of COVID-19 patient attention [41]. Despite leadership changes during the pandemic, which were described as a double crisis both in health and governance in Bolivia by [42], there was a gradual shift toward more decentralized services and improved coordination. Early centralized approaches may have reduced the health system’s ability to mobilize resources and prioritize testing and inpatient care needs during subsequent surges. Another pre-pandemic condition important to consider when evaluating our results is the fragmentation of Bolivia’s healthcare system, particularly between the two public subsectors. Fragmentation of the system has been identified as a barrier even in developed countries such as Italy in the context of the pandemic response [43]. Given the unequal fragmentation of the Bolivian healthcare system in terms of resources and population, the initial centralized approach could have also exacerbated disparities between the health system sectors by not allowing for homogeneous early resource development or future surge preparation.

Consistent with other reports, the results show that, due to already limited staffing levels before the COVID-19 pandemic, even compared to other similar countries in the region, human resources in Bolivia and Cochabamba decreased significantly during the pandemic [44]. In response, the health system had to introduce new laws and regulations to enable short-term hires and expand staffing capacity. Importantly, this study illustrates how the introduction of a unique stay-at-home benefit for public employees in a high-risk group was widely utilized by health staff and, at least in part, used to avoid working in the public health sector while still receiving their salary and benefits, and causing severe staff attrition when they were most needed. Other reports on pandemic policies that either increased or reduced workforce capacity during the COVID-19 pandemic have focused on staffing challenges, such as those of Canadian nursing homes, where immigration laws seem to have been partly responsible for staff attrition [8]. These findings highlight the importance of careful consideration when implementing policies and the necessity of considering potential human resource impacts, including staff attrition. In Bolivia, the stay-at-home policy described was intended to protect public staff in high-risk groups in many areas. However, applying it to healthcare staff created a problematic situation in addition to the already deficient staffing levels pre-pandemic. Other reports in the Bolivian context have analyzed workforce deficiencies during the pandemic [44, 45]. Previous research has not identified the stay-at-home policy, which, according to our study, led to worsening staff attrition. By identifying these policies and their implications, our study provides valuable insights to inform future policymaking strategies in Bolivia and other LMICs, even outside Latin America.

The results also illustrated other service delivery challenges that spurred innovation through increased flexibility, adaptation, the use of telemedicine, and other traditional medicine services. This finding aligns with other pandemic-related research, which describes the expansion of the Bolivian telemedicine program [46], as well as reports of similar initiatives in the US, Lebanon, and Kenya [47–49]. The lack of infrastructure and equipment pushed the healthcare sector in Bolivia to develop innovative and adaptive strategies. Specifically, system leaders had to find creative solutions to address overwhelming patient loads and critical supply shortages. This resonates with many innovations in other LMICs, such as South Africa [50], where PPE preservation and reutilization strategies were applied. In addition, developed nations such as Canada [51] used the challenges experienced to build recommendations for better supply chain management. The private sector played a crucial role, providing much-needed space and equipment. These public-private partnerships have also been described in other LMICS, such as the Congo, Nigeria, Senegal, Uganda [11], and India during the pandemic response [12]. There, public‒private partnerships were instrumental in producing supplies and tests, promoting adherence to preventive policies, and even expanding the capacity for healthcare service delivery. From these experiences, we can conclude that increases in public‒private partnerships for the domestic production of emergency supplies should be strengthened, formalized, and expanded to create sustainable cross-sector collaborations that could enhance health systems.

The pandemic also accelerated the adoption of telemedicine and other nontraditional healthcare services in the country, similar to other parts of the world [52]. However, like conventional medicine, telemedicine can only complement, not replace, the need for adequately staffed and equipped healthcare facilities. This was described, for example, in the heterogeneity of telemedicine applications across different specialties, as expected, with some being easier to adapt to this service delivery system than others [53]. In the case of Bolivia, the increased adoption of telemedicine was an expansion of tools already used by the population for their healthcare delivery needs. The use or expansion of nontraditional or non-occidental medicine was also an important element to consider in the Bolivian context. In other contexts, research has reported the use of traditional Chinese medicine [54] and traditional Vietnamese medicine [55]. Both for COVID-19 during the pandemic and for preventing and treating mild cases in parallel to conventional medical treatments. In Bolivia, the BMoH even provided guidelines for using traditional medicine to treat COVID-19 [56]. This was an important component of the community’s response to the pandemic. The extensive use of these treatments shows promise but is also associated with spurious, unstudied, and unconventional treatments for occidental or traditional medicines, such as ivermectin and chlorine dioxide, for COVID-19 treatment. The use of these two nonconventional treatments has been described in research in Peru [57] but remains unexplored in the Bolivian context.

Regarding the implications of this work for the mortality and morbidity of COVID-19 during this period, we can infer that certain elements may have contributed to increased mortality or morbidity, while others may have mitigated it. The timing and the application mechanisms of these strategies may have influenced both outcomes. Centralization may have initially had a positive impact, but when saturated services became insufficient, it would have reduced the health system’s capacity to provide treatment to the affected population. The attrition of health staff may have had adverse effects on the capacity to treat patients, and the resulting short-term hires may have mitigated the magnitude of this adverse effect. On the other hand, innovation, traditional medicine, and telemedicine may have had positive effects, particularly on access and morbidity associated with the condition.

### Methodological considerations

This is the first study exploring the health system’s response to the COVID-19 pandemic from the perspectives of policymakers and managers in Cochabamba, Bolivia. The research team was familiar with some of the realities explored, and most of our team is fluent in both English and Spanish. Our pilot interview informed the development of the interview guide. We took steps from initial descriptive codes to more conceptual categories, engaging in a systematic coding process. Codes were developed in English, whereas transcripts and meaning units remained in their original language, Spanish, to preserve linguistic nuance. We consider ourselves as active participants in data collection and interpretation. Therefore, we adopted a cyclic, constructivist epistemological stance and employed an abductive approach to thematic analysis, with each step built upon and refined from the previous one, generating reflections, memos, and insights that shaped the core conclusions. This approach was considered appropriate for this study, as it allowed for flexibility in analytic thematization and focused our results on the responses of health system leaders to the pandemic. We progressed from concrete codes to more abstract thematic constructs through constant comparison and analysis. The final themes were developed from the data and interpreted by sensitizing concepts from relevant health system frameworks. This approach supported theoretically informed but grounded analysis results.

The sampling process was designed to enhance transferability, with a purposive selection of key informants across the private and public health system subsectors (public and social security). While our institutional affiliation facilitated access to specific high-level stakeholders, it is possible that our participants may have exhibited some level of social desirability bias in their responses. Additionally, a potential recall bias, given the time elapsed between the interviews and the COVID-19 experiences, may have been present. The participants may have expected the research team to share opinions in line with what they perceived as a consensus on COVID-19-related policies or responses, which may have inadvertently limited participation from more critical or opposing voices. We sought to mitigate this by conducting interviews across diverse roles and perspectives and fostering a safe and open discussion space.

We employed reflexive memos, notes, and iterative team discussions throughout the study to enhance analytical depth and consistency. While we approached the data openly, we recognize that both researchers and participants, as trained professionals, may have drawn on shared assumptions and prior knowledge about systemic deficiencies in Bolivia’s health system. We actively reflected on these dynamics to ensure that they enriched rather than constrained our interpretations.

### Conclusions and recommendations

This study provides valuable contributions to the literature by exploring the responses of health system leaders to pandemic emergencies. The governance, human resources, and service delivery challenges described are unique to this context but highlight challenges commonly affecting other LMIC countries in this region [44, 58]. The COVID-19 pandemic seems to have exacerbated preexisting weaknesses within the Bolivian healthcare system, making fragmentation, inequality, and lack of preparedness more visible. Despite these challenges, the system demonstrated remarkable adaptability and innovation. The present study offers important insights into previously underreported aspects in this context, which should be considered when planning future emergency responses to achieve a more resilient system.

Several recommendations can be made based on the results. First, coordination needs to be improved, and communication between the fragmented health sector and society stakeholders should be strengthened, as political disputes and initial centralization hinder responsiveness. Second, health workers should be granted special protected status as essential personnel to prevent staff attrition during emergencies, and regulations should guarantee that service levels never decline from pre-emergency periods. Finally, strengthening public‒private partnerships can ease resource bottlenecks and improve service delivery, given the dependency of the Bolivian health system on other countries’ production of these resources.

## Supplementary Information

Below is the link to the electronic supplementary material.

Supplementary Material 1

Supplementary Material 2

## Data Availability

The data from transcripts used and analyzed in the current study are available from the corresponding author upon reasonable request.

## Abbreviations

COVID-19: (Coronavirus Disease Of 2019)
LMIC: (Lower-Middle-Income Country)
Bmoh: (Bolivian Ministry Of Health)
Ngos: (Non-Governmental Organizations)
Icus: (Intensive Care Units)
PPE: (Personal Protective Equipment)
CPAP: (Positive Airway Pressure)
