# Supplementary material for: Exploring the health system response to the COVID-19 pandemic in Cochabamba, Bolivia: a qualitative study with policymakers and managers
Source: BMC Health Serv Res. 2025 Sep 15;25:1200. doi: 10.1186/s12913-025-13483-1 (PMC12439360; doi:10.1186/s12913-025-13483-1)
Supplement: Supplementary file 1 — Supplementary Material 1 [file 12913_2025_13483_MOESM1_ESM.docx]

| **Individual Qualitative Instrument (English)**  **(Estimated time of the interview 30min – 1hr)**  **Semi-structured interviews**  *(These are only guides to prompt the interviewer and interviewee to adhere to the topic. Please feel free to add the themes and concerns as you see fit)*  **Introduction (please adapt as you see fit):**  I am ………………………… from the ……………………….. and I am approaching you to conduct a study related to the COVID-19 pandemic. As you know, the pandemic has strained our health system; I would like to know your perspectives as a manager or policymaker regarding the healthcare personnel allocation and resource management during the pandemic.  There are no consequences attached to participating in this study. In addition, if you agree, I would like to audio-record this interview for transcription and analysis. I would like to inform you that this interview will be anonymized, the information you will provide will be completely confidential, and the storage of any all data including recordings will be done at a secure server of our institution and with restricted access to the study team. You can feel free to drop out of the interview at any time and you don’t have to give me reasons for dropping out.  If you agree, I would like to start with the begin asking you some questions regarding your role during the pandemic….  **AUDIO FILE NAME:** | | |
| --- | --- | --- |
| **SECTION-I: Demographic and role characteristics** | | |
| **Socio-demographic characteristics of respondents** | 1. Age |  |
|  | 2. Gender |  |
|  | 3. Region (of their role) |  |
|  | 4. Municipality (of their role) |  |
|  | 5. Institution |  |
|  | 7. Type of institution (public, social security, or private) |  |
|  | 8. Position |  |
|  | 9. Years of experience in this role |  |
|  | 10. If not currently, period of experience in this role |  |
|  | 11. Level of management (e.g.: National, Regional, etc) |  |
|  | 12. Education level |  |
| **SECTION-II: Open questions** | | |
| **Themes** | **Guide** | |
| COVID-19  public health  Management | 1. What is your professional opinion of the current COVID-19 pandemic situation? 2. Is the current preparedness at Cochabamba/Your Institution adequate to control the infection and why? 3. Do you think the public health measures taken by the government where helpful for the control of COVID-19 infections and why? 4. How did the government preparedness and public health measures changed since the beginning of the pandemic until today? | |
| Characteristics of the Bolivian Health  System | 1. In your opinion, was our (Bolivian) healthcare system prepared to manage the COVID-19 pandemic? Please elaborate. 2. In terms of infrastructure and resources, what are the limitations and challenges that you have experienced in managing COVID-19 cases in Bolivia? 3. What kind of things do you think could be done by the Bolivian government to overcome those challenges? Have any of them been addressed already? | |
| Allocation of Human resources | 1. How did the frontline health workers in Cochabamba/your institution allocated? 2. Do you think we had or have adequate numbers of frontline health workers in Cochabamba/your institution to handle the COVID-19 pandemic and why? 3. Do you think health workers in Cochabamba/your institution are prepared with adequate infection control measures? 4. Can you talk about the impact of the outbreak on frontline health workers (prompts: fear of being infected, financial hardship, increased stress, and workload)? 5. Have you faced discrimination or stigma from your friends or community because of your role in the COVID-19 pandemic? | |
| Allocation of equipment/resources management (space, equipment, and facilities) | 1. How was medical equipment allocated in Cochabamba/your institution? 2. Do you think Cochabamba/your institution had adequate medical equipment to take care of COVID-19 cases and why? 3. What equipment is lacking? And why do you think they are lacking? 4. Does Cochabamba/ your institution had adequate space/area/beds allocated? especially for COVID 19 cases, isolation wards, ICU etc. to provide care to COVID-19 cases and why? 5. How were space/area/beds allocated in Cochabamba/your institution during the COVID-19 pandemic and why? | |
| Recommendations/  Way forward | 1. What are your projections for the near future for COVID-19 in Bolivia? 2. Could you please use this opportunity to give recommendations in regard to the limitations? 3. What should be the way forward in terms of preparing the health system and health workers to fight against similar epidemics or emergencies in the future? | |
| Other areas of the response not explored in this instrument | 1. Is there something else that you think is important to discuss that I haven’t asked you about? | |

| The End |
| --- |
